# Cell cycle arrest and p53 prevent ON-target megabase-scale rearrangements induced by CRISPR-Cas9

**DOI:** 10.1038/s41467-023-39632-w

**Published:** 2023-07-10

**Authors:** G. Cullot, J. Boutin, S. Fayet, F. Prat, J. Rosier, D. Cappellen, I. Lamrissi, P. Pennamen, J. Bouron, S. Amintas, C. Thibault, I. Moranvillier, E. Laharanne, J. P. Merlio, V. Guyonnet-Duperat, J. M. Blouin, E. Richard, S. Dabernat, F. Moreau-Gaudry, A. Bedel

**Affiliations:** 1 Bordeaux University, INSERM, BRIC, U1312, F-33000 Bordeaux, France; 2 CHU de Bordeaux, Biochemistry Laboratory, F-33000 Bordeaux, France; 3 CHU de Bordeaux, Tumor Biology and Tumor Bank Laboratory, F-33000 Bordeaux, France; 4 CHU de Bordeaux, department of medical genetics, F-33000 Bordeaux, France; 5 Vect’UB, vectorology platform, INSERM US 005—CNRS UAR 3427-TBM-Core, Bordeaux university, Bordeaux, France

**Keywords:** Targeted gene repair, Molecular medicine, CRISPR-Cas9 genome editing, DNA damage response

## Abstract

The CRISPR-Cas9 system has revolutionized our ability to precisely modify the genome and has led to gene editing in clinical applications. Comprehensive analysis of gene editing products at the targeted cut-site has revealed a complex spectrum of outcomes. ON-target genotoxicity is underestimated with standard PCR-based methods and necessitates appropriate and more sensitive detection methods. Here, we present two complementary Fluorescence-Assisted Megabase-scale Rearrangements Detection (FAMReD) systems that enable the detection, quantification, and cell sorting of edited cells with megabase-scale loss of heterozygosity (LOH). These tools reveal rare complex chromosomal rearrangements caused by Cas9-nuclease and show that LOH frequency depends on cell division rate during editing and p53 status. Cell cycle arrest during editing suppresses the occurrence of LOH without compromising editing. These data are confirmed in human stem/progenitor cells, suggesting that clinical trials should consider p53 status and cell proliferation rate during editing to limit this risk by designing safer protocols.

## Abstract (web summary)

ON-target genotoxicity in gene editing is generally underestimated. Here the authors report Fluorescence-Assisted Megabase-scale Rearrangements Detection (FAMReD) systems to detect and characterize rare large loss of heterozygosity: they show that ON-target genotoxicity can be prevented by p53 and cell cycle arrest.

## Introduction

Targeted nucleases, and in particular the CRISPR-Cas9 system, are a breakthrough that has propelled gene therapy into a new era^1–4^. Important advances are illustrated by several ongoing preclinical and clinical studies in fields such as immunotherapy, virology, and monogenic diseases. Nevertheless, a major concern is the potential genotoxicity of DNA double-strand breaks (DSB), which arise from incorrect or ineffective DNA repair and DNA damage response. The risk of genomic instability seems to be the Achilles heel of CRISPR–Cas9. Detailed genotyping of edited cells revealed that the full spectrum of Cas9-induced outcomes might be more complex than the sole induction of insertion and deletions InDels at the targeted locus (ON-target genotoxicity)^5^. Kilobase-scale deletions, inversions and insertions have been reported in primary murine and human cells^6–10^. Chromosome truncations associated with megabase-scale loss-of-heterozygosity with copy losses (CL-LOH) have been reported not only in cancer cell lines^10–12^ but also in p53-proficient human induced pluripotent stem cells (hiPSC) and embryos^9,13^. Terminal megabase-scale copy-neutral LOH (CN-LOH) without loss of genetic material have also been observed in cancer cell lines^14^, human embryonic stem cells (hESC) and hiPSC^9–15^. Gene editing of human hematopoietic stem/progenitor cells (HSPC) at the beta-globin cluster also gave rise to CN-LOH, associated with an abnormal methylation profile of 11p15 imprinting centers (disomy of maternal or paternal allele) with altered transcriptional activity^16^. Finally, chromothripsis^17^ and entire chromosome losses^13,18–21^ can occur in response to a single DSB.

Current molecular tools for identifying gene-editing products mostly rely on PCR amplification, which does not detect large genetic events. Cytogenetic methods are more appropriate but often lack sensitivity to detect rare events in polyclonal populations. The issue of the detection sensitivity threshold can be circumvented by using clonal analysis but at the cost of a tedious, time-consuming, expensive procedure that is incompatible with high-throughput analysis.

Here, we present two complementary Fluorescence-Assisted Megabase-scale Rearrangements Detection (FAMReD) systems that detect, quantify, and precisely characterize rare extra-large LOH in edited live cells by cell sorting. We show that the DSB location does not influence LOH frequency and that diverse megabase-scale rearrangements (including terminal CL-LOH, CN-LOH and/or duplication) with a majority of CN-LOH are present in cells. These tools offer highly sensitive readouts to decipher the short-term (mFAMReD) and long-term (hFAMReD) risk and to find solutions to limit it. We report that LOH frequency increases with p53 inactivation while low cell division drastically reduces this risk. Understanding the mechanisms involved in ON-target genotoxicity is a first step to limit them and to improve the safety of CRISPR-based clinical applications.

## Results

### Detection of megabase-scale rearrangements in human cells: the hFAMReD system

**Fig. 1:**
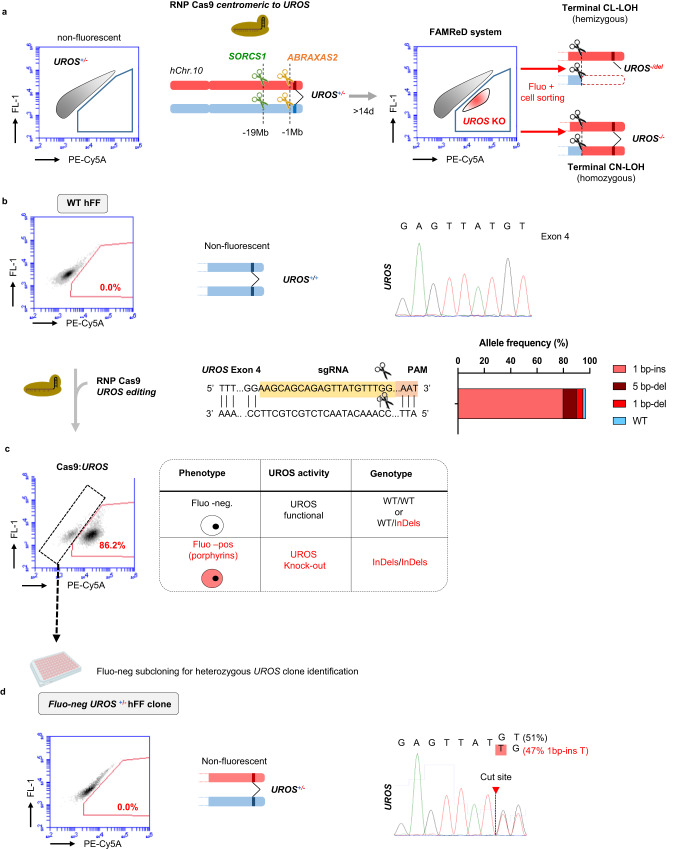
hFAMReD. **a** hFAMReD description. Non-fluorescent *UROS*^+/-^ fibroblasts are transfected with a RNP Cas nuclease targeting different loci centromeric to *UROS*. Upon telomeric LOH encompassing *UROS* (CL-LOH or CN-LOH), loss of UROS function induces appearance of fluorescence by porphyrin accumulation detected by flow cytometry. Fluorescent cells are analyzed at least 14 days after transfection to be sorted and characterized. **b** WT hFFs are non-fluorescent. Sanger sequencing and schema of *UROS*^WT^. Generation of *UROS*^+/-^ hFF by RNP Cas9:*UROS* transfection. Representation of *UROS* sequence targeted by Cas9 RNP. gRNA location in yellow. PAM in red. Scissors represent gRNAs cut-sites. Analysis of most common observed alleles (with frequencies ≥1%) by ICE software. **c** non-fluorescent cell sorting and subcloning to screen for *UROS*^*+/-*^ fibroblasts. **d** Characterization of one *UROS*^+/-^ hFF clone used in hFAMReD system. Illustrative analysis of non-fluorescent cells by cytometry. Sanger sequencing reveals a heterozygous +1 ( + T) insertion in *UROS*. CL-LOH: copy-loss LOH (deletion), CN-LOH: copy-neutral LOH.

We developed a sensitive approach to detect and quantify LOH induced by nucleases on human Chr10q and to sort live edited cells for their in-depth characterization. Briefly, hFAMReD relies on a cell phenotype switch, from non-fluorescent to fluorescent, induced by a megabase-scale LOH (Fig. 1a). This switch is due to the accumulation of fluorescent red porphyrins occurring in UROS-deficient cells^22^, which is readily detectable by flow cytometry upon ALA (5-amino-levulinic acid) precursor exposure (Supplementary Fig. 1).

To create this system, immortalized human foreskin fibroblasts (hFFs) were first edited with a ribonucleoprotein (RNP) Cas9:gRNA complex targeting *UROS* exon 4 in 10q26.2 to generate a cell line heterozygous for a loss-of-function variant in the *UROS* gene (*UROS*^+/-^) located 7.5 Mb from the telomere (Fig. 1b–d). Editing efficiency was high (98% of InDels), resulting in 86.2% of fluorescent cells. To select the *UROS*^+/-^ hFF clone (with preserved normal heme biosynthesis), we sorted and subcloned the non-fluorescent fraction (Fig. 1c). We selected and amplified one *UROS*^+/-^ hFF clone and used it thereafter (Fig. 1d).

In this study, we used the Cas9 nuclease to target loci centromeric to *UROS*, located at least one megabase from *UROS* and 8.5 Mb from the telomere (Fig. 1a). The megabase-scale LOH (copy-loss or copy-neutral) thus induced can disrupt the remaining functional *UROS* allele. The fluorescent switch is then activated without affecting their proliferation (Supplementary Fig. 2), and the persistent rearranged LOH^+^ cells can be detected, quantified and sorted for in-depth analyses (and Fig. 1a).

### LOH detection in fibroblasts (hFFs)

**Fig. 2:**
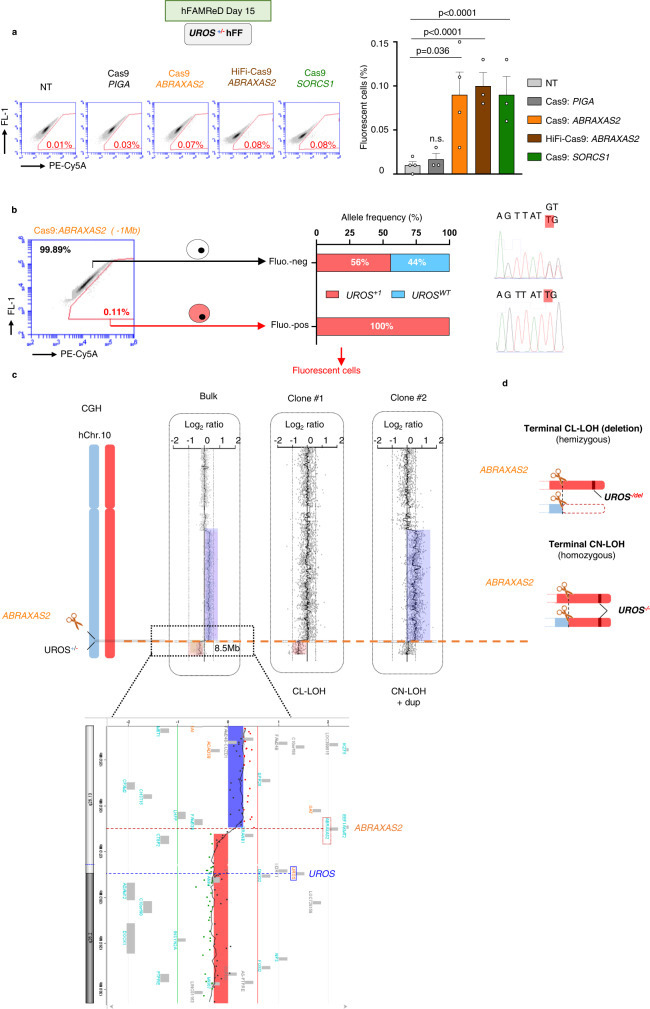
hFAMReD reveals large rearrangements in hFFs after ABRAXAS2 editing. **a** Representative cytometry analysis of fluorescent cells (PE-Cy5A^+^) and associated quantifications in *n* = 3 independent experiments (*n* = 4 for *ABRAXAS2* editing) (mean ± SD). *PIGA* targeting (negative control, dark gray, located on Chr.X, InDels > 90%), *ABRAXAS2* with classical (orange) and HiFi-Cas9 (brown) (InDels > 50%) and *SORCS1* (green, InDels > 60%) are compared to non-transfected (NT, light grey) UROS^+/-^ hFFs. **b** After Cas9:*ABRAXAS2* editing, fluorescent-positive and negative cell sorting for *UROS* Sanger sequencing and LOH confirmation at *UROS*. **c** array CGH of fluorescent-positive cell bulk (left) and two fluorescent-positive clones, #1 and #2 (right). Deletions in pink, duplications (dup) in blue. **d** Illustration of megabase-scale rearrangements detected by FAMReD in hFFs after *ABRAXAS2* editing. Two types of LOH, CN-LOH and CL-LOH. Anova test used to compare multiple groups, Mann–Whitney test used to compare two groups. Source data are provided as a Source Data file.

In our previous study, fluorescence in situ hybridization (FISH) did not reveal a significant level of megabase-scale CL-LOH (truncation) in edited WT (p53-proficient) hFFs^11^. However, FISH sensitivity may not be sufficient to detect rare deletions and cannot detect CN-LOH. Here, we targeted the same chromosome arm (Chr10q) and used hFAMReD to determine whether LOH occurred at low frequency in hFFs. By targeting *ABRAXAS2* (10q26.13) 1 Mb centromeric to *UROS* (Fig. 1a), we observed a slight increase in the level of fluorescent cells (PE-CyA5^+^: 0.09 ± 0.05%) 15 days after editing, suggesting the occurrence of LOH at the *UROS* locus. Similar results were obtained using the high fidelity HiFi-Cas9 nuclease (0.10 ± 0.02%), which was previously reported to have a reduced off-target activity^23^. None of the Top-10 predicted off-target sites are located on Chr10q (Supplementary Table 1), thus ruling out an off-target confounding factor as a source of unwanted editing. In contrast, fluorescent cells were absent without transfection and when *PIGA*, localized on ChrX, was targeted (0.01 ± 0.01% and 0.02 ± 0.01%, respectively (Fig. 2a). These data show that CRISPR-Cas9 nuclease induces megabase-scale LOH in p53-proficient fibroblasts at a rate of around one cell with extra-large rearrangement per 1000 edited cells, which is undetectable by FISH. Additionally, no significant increase in fluorescent cell count was evidenced using the catalytically inactive deadCas. Using the Cas9^D10A^ nickase, the LOH rate was not significantly different from controls (Supplementary Fig. 3). We cannot exclude the possibility of rare LOH with single-strand breaks (SSB) induced by nickase (as observed in ref. ^11^).

We next sorted the fluorescent cells (Fig. 2b) and genotyped *UROS*. Only the mutated *UROS* allele was detected, suggesting that all fluorescent cells had lost the *UROS* wild-type (*UROS*^wt^) allele. Conversely, in the non-fluorescent fraction, cells exhibited a *UROS*^+/-^ heterozygous status comparable to that observed in the initial cell line.

To better delineate and map the DSB-induced LOH, we performed array comparative genomic hybridization (aCGH) on the sorted fluorescent cell fraction after *ABRAXAS2* targeting (Fig. 2c, “bulk”). We detected an interstitial 72 Mb duplication with the distal breakpoint located at the cut-site, and a Log2ratio of +0.3 suggestive of a mosaicism, i.e., about 46% of cells carrying interstitial genomic duplication. We also observed an 8.5 Mb terminal deletion with the breakpoint at the cut-site and a Log2ratio of –0.28 suggestive of a mosaicism with 36% of the cells harboring terminal deletion, suggesting the predominant presence of CN-LOH in addition to CL-LOH. The analysis of two fluorescent clones confirmed the presence of both types of LOH (Fig. 2c, clones): clone #1 presented a terminal CL-LOH starting from the cut-site while clone #2 had a CN-LOH starting from the cut-site, with an extra-large interstitial 10q duplication detected in the bulk analysis. Notably, these genomic abnormalities were not detectable by aCGH or by single nucleotide polymorphism (SNP) array without cell sorting (without hFAMReD LOH enrichment, Supplementary Fig. 4).

Collectively, these results suggest the occurrence of DSB-induced megabase-scale LOH in edited hFFs and confirm the high sensitivity of the hFAMReD system to detect and isolate them for precise characterization (Fig. 2d). Importantly, LOH^+^ cells sorted after day 15 were long-term rearranged cells. They persisted for at least 40 days, without selective advantage or disadvantage (Supplementary Fig. 2).

**Fig. 3:**
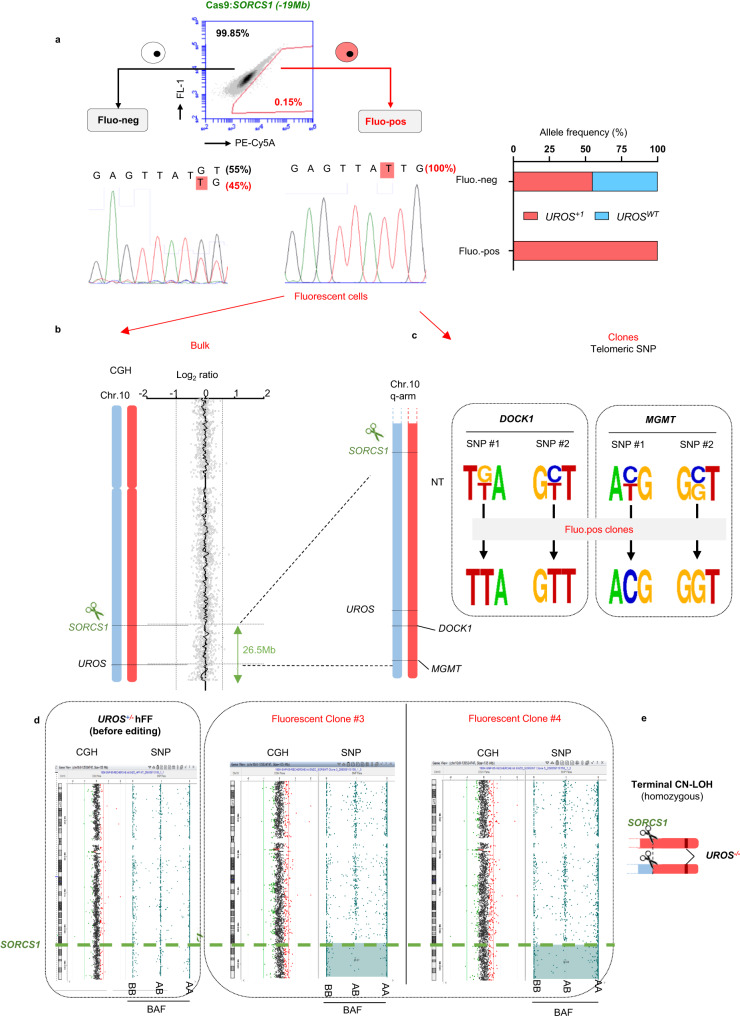
hFAMReD reveals large rearrangements in hFFs after SORCS1 editing. **a** After Cas9:*SORCS1* editing, fluorescent-positive and negative cell sorting for *UROS* Sanger sequencing and LOH confirmation. **b** Array cGH of fluorescent-positive cell bulk showing no copy number variation. **c** SNP Sanger sequencing of *DOCK1* (located at +1.4 Mb from *UROS*) and *MGMT* (+3.4 Mb) in non-transfected (NT) cells and in fluorescent-positive clones. **d** Array CGH and SNP array of cells before editing and two fluorescent-positive clones. Log_2_ratio and BAF (B-allele frequency) in control-hFFs (before editing) and two edited fluorescent clones (#3 and #4). Regions with loss of heterozygous AB alleles in blue. **e** Illustration of megabase-scale rearrangement detected by hFAMReD in hFFs after *SORCS1* editing (CN-LOH). Source data are provided as a Source Data file.

We then focused on *SORCS1*, which is 19 Mb centromeric to *UROS*. For this targeted locus, we obtained a similar level of fluorescent cells (0.09% ± 0.02, Fig. 2a) compared to the *ABRAXAS2*-edited hFFs. Again, the sorted fluorescent cells only displayed the *UROS* loss-of-function variant allele (Fig. 3a). aCGH analysis on *SORCS1*-edited fluorescent cells did not reveal any copy number variation (CNV), which was suggestive of a loss of UROS function by CN-LOH (Fig. 3b, “bulk”). To confirm the presence of extra-large CN-LOH, we identified and sequenced SNPs in *DOCK1* and *MGMT*, which are both located between *UROS* and the telomere. While the parental cell line was heterozygous for the tested SNPs, 6/6 fluorescent screened clones were homozygous for these SNPs (Fig. 3c), suggestive of a CN-LOH from *SORCS1* to the telomere. A combined aCGH/SNP array in the same clones demonstrated the absence of deletion and the presence of a 26.5 Mb terminal CN-LOH, starting from the cut-site (Fig. 3d).

**Fig. 4:**
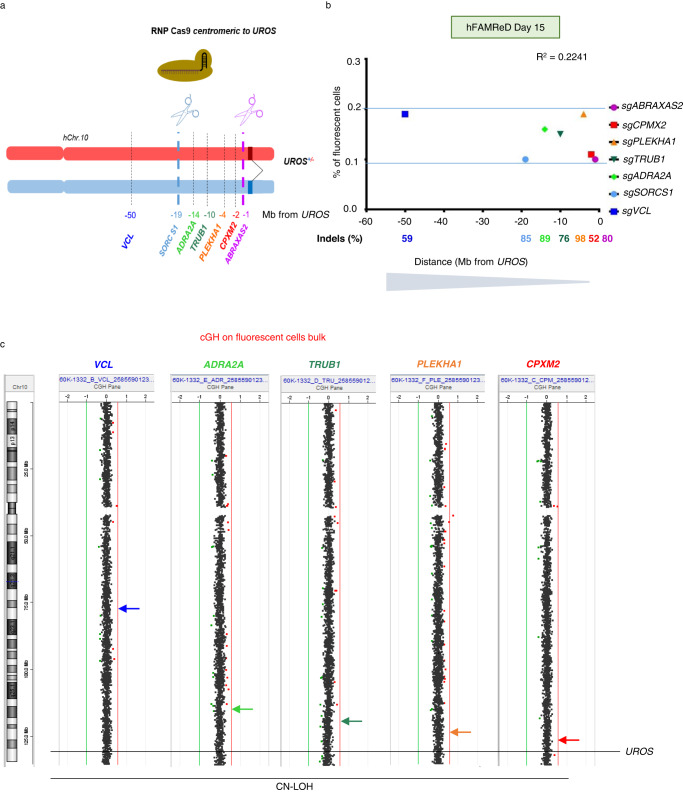
Absence of correlation between the LOH frequency and the distance to the telomere. **a** Schema of gRNAs location on hChr10q, with various distances between the gRNA and *UROS*. **b** Absence of correlation between LOH frequency and distance to *UROS* (Pearson R^2^ = 0.22). All indels were >50%. **c** Array CGH of fluorescent-positive cell bulk showing CN-LOH when gRNA is farther than 1 Mb to *UROS*. Source data are provided as a Source Data file.

To establish whether the location of DSB affects the frequency and the LOH type, we next targeted hChr10 with five other gRNA targeting *CPXM2, PLEKHA1, TRUB1, ADRA2A and VCL respectively* 2, 4, 10, 14 and 50 Mb centromeric to *UROS* (Fig. 4a). For each guide RNA, we obtained a high InDel rate (>50%) and very similar LOH frequencies (from 0.1 to 0.2% of fluorescent cells in WT hFFs, even with the farthest away cut). The Pearson correlation coefficient was low (R^2^ = 0.22), demonstrating the absence of correlation between the distance to the telomere and the LOH frequency (Fig. 4b). Interestingly, aCGH of the fluorescent cell bulk for each additive gRNA was normal (Fig. 4c), suggesting that CN-LOH are predominant. When we targeted loci more than 2 Mb away from *UROS*, CL-LOH were under the aCGH limit of detection (10–20%). Altogether, these results seem to indicate that the targeted locus does not influence the frequency of the total LOH rate but might modify the relative proportion of CN-LOH and CL-LOH.

### p53 inhibition increases DSB-induced LOH frequency

**Fig. 5:**
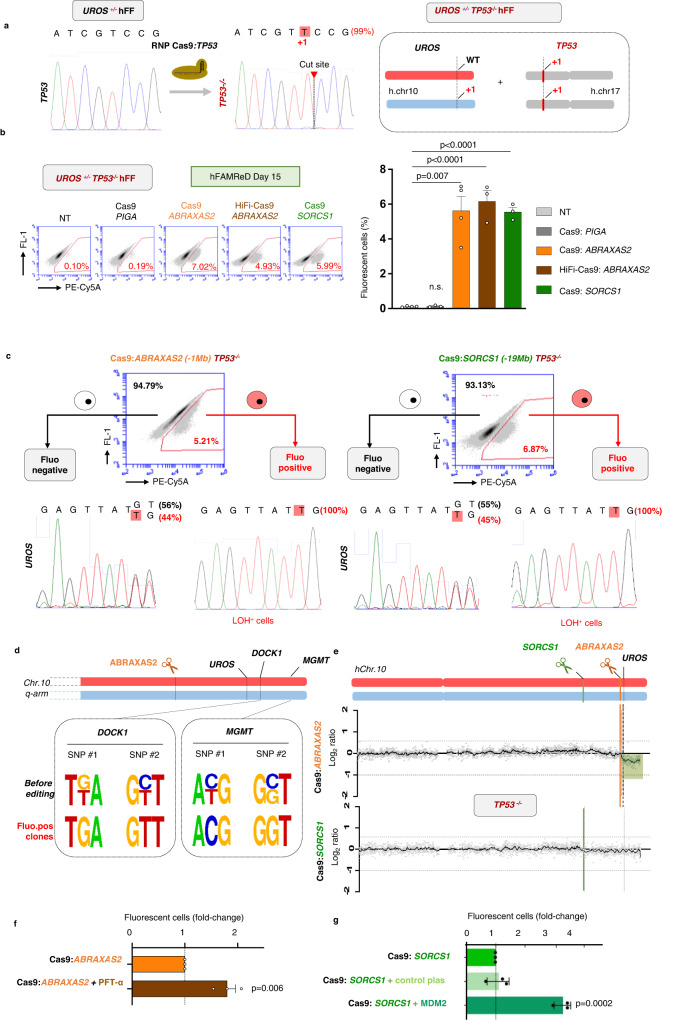
TP53 inactivation increases LOH frequency. **a** *TP53* inactivation by CRISPR-Cas9 nuclease transfection. Sanger sequencing and ICE software to confirm InDels in a homozygous edited clone. **b** Representative cytometry analysis of fluorescent cells induced by editing. Quantifications in *n* = 3 independent experiments (*n* = 4 for non-transfected (NT) and *ABRAXAS2* editing) (mean ± SD). *PIGA* targeting (negative control, dark grey, InDels > 90%), *ABRAXAS2 targeting* (InDels > 50%) with classical (orange) or HiFi-Cas9 (brown), and *SORCS1* targeting (green, InDels > 50%) are compared to NT (light grey) (*UROS*^+/-^*TP53*^-/-^ hFFs. **c** After Cas9:*ABRAXAS2* editing (left) or Cas9: *SORCS1* editing (right) in *TP53*^-/-^ cells, fluorescent-positive and negative cell sorting for *UROS* Sanger sequencing and LOH confirmation. **d** Illustrative SNP Sanger sequencing of *DOCK1* (+1.4 Mb from *UROS*) and *MGMT* (+3.4 Mb) in cells before editing and in six fluorescent clones after *ABRAXAS2* editing. **e** Array-CGH of fluorescent-positive cell bulks after *ABRAXAS2* and *SORCS1* editing. Deletion in green. **f** Fluorescent cell quantification after Cas9:*ABRAXAS2* editing, with (brown) or without (orange) pifithrin-alpha exposure (30 µM, 15 h before transfection and for 5 days after transfection, *n* = 3 independent experiments, mean ± SD). **g** Fluorescent cell quantification after Cas9:*SORCS1* editing, with MDM2 plasmid (dark green) or empty pcDNA3 control plasmid (light green) or without any plasmid (green) (*n* = 3 independent experiments, mean ± SD). Anova test (two-sided) used to compare multiple groups, Mann–Whitney test (two-sided) used to compare two groups. Source data are provided as a Source Data file.

p53 plays an essential role in DNA damage sensing and repair^24,25^ and is activated by CRISPR-induced DSB^26–29^. We previously demonstrated the strong involvement of p53 in chromosomal instability induced by CRISPR-Cas9 with a dramatic increase in megabase-scale CL-LOH (truncation) in p53-deficient hFFs^11^. However, the impact of p53 deficiency on the global LOH rate, including CN-LOH and CL-LOH, is still unknown. *TP53* was inactivated with an RNP Cas9:gRNA targeting *TP53* in the hFAMReD *UROS*^+/-^ hFFs, using the same RNP previously reported^11^. We selected a clone homozygous for a loss of function variant in *TP53* (*TP53*^-/-^, Fig. 5a), with a normal karyotype (Supplementary Fig. 5). In p53^-/-^ fibroblasts, *ABRAXAS2 and SORCS1* targeting (located 1 Mb and 19 Mb centromeric to *UROS*, respectively) led to the occurrence of 5.6% ± 0.8 and 5.56% ± 0.3 of fluorescent cells, respectively (Fig. 5b), a 60-fold increase compared to p53-proficient-hFFs (compared to WT cells, Fig. 2b). Minimal levels of fluorescent cells were observed in non-transfected cells and in cells edited with an RNP targeting *PIGA* (located on ChrX) (Fig. 5b). Again, HiFi-Cas9 editing did not decrease the rate of fluorescent cells (6.2 ± 0.6%, Fig. 5b) and LOH frequencies were independent of distance-to-telomere and of OFF-target events.

After editing of either *ABRAXAS2* or *SORCS1*, sequencing of fluorescent cells confirmed the loss of the *UROS*^WT^ allele (Fig. 5c), and SNP allelic losses for *DOCK1* and *MGMT* for *ABRAXAS2* (Fig. 5d), indicating that the fluorescent cells harbored LOH encompassing *URO*S and genes near to the telomere. aCGH of polyclonal fluorescent *ABRAXAS2*-edited cells revealed a mosaic 10q26.13qter deletion (Log2ratio: −0.33, 41% of cells) suggestive of a mix of CL-LOH and CN-LOH (Fig. 5e, up). In contrast, aCGH was almost normal after *SORCS1* targeting, suggestive of rare deletions (Log2ratio −0.08, 10% of alleles) and a high prevalence of CN-LOH (Fig. 5e, bottom).

Transient p53 inhibition during editing is thought to limit apoptosis, increase editing efficiency and hematopoietic-edited cell engraftment and therefore could be of clinical interest^28,30^. We tested the effect of the transient inhibition of p53 on LOH frequency using pifithrin-alpha and MDM2 plasmid. Both transient p53 inhibitions increased LOH frequency (1.8-fold, Fig. 5f and 3.6-fold, Fig. 5g). Notably, this increase in LOH was lower than that observed by the long-term inactivation of p53. Taken together, hFAMReD precisely quantifies the increased risk of megabase-scale LOH in p53-deficient cells compared to p53-proficient cells. These results confirm the role of p53 in maintaining genome stability after a CRISPR-Cas9 DSB. p53 deficiency is involved not only in CL-LOH (truncations) but also in copy neutral LOH induced by Cas9 nuclease.

### Cell cycle drives DSB-induced megabase-scale ON-target LOH

**Fig. 6:**
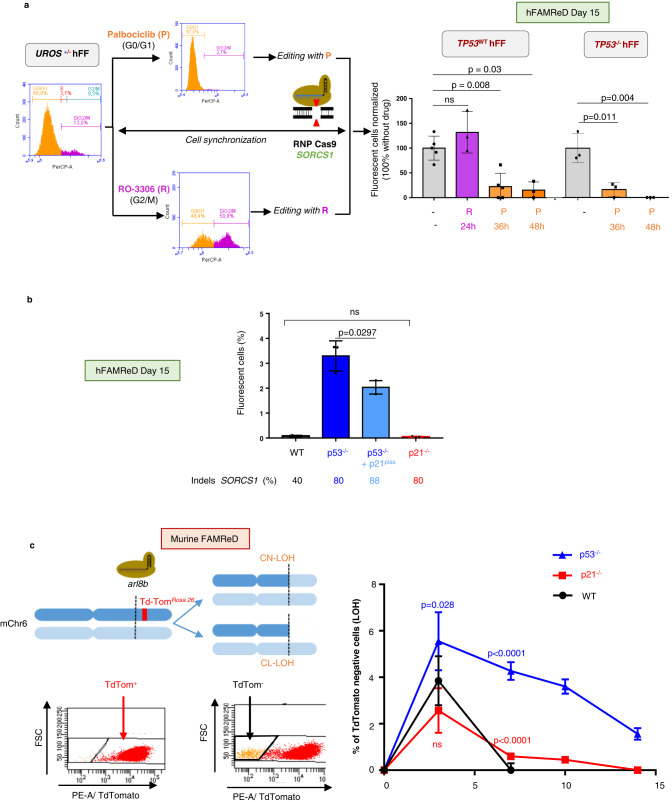
FAMReDs show that CDK modulation affects Cas9-induced LOH frequency. **a** Cells (*TP53*^WT^ and *TP53*^*-*/-^) were exposed to palbociclib or RO-3306 to synchronize cells in G0/G1 (orange) or G2/M (purple) phases respectively before and during editing. Cell cycle analysis by flow cytometry (PerCP FACS plots corresponds to propidium iodide) at day 0 during CRISPR event. Fluorescent cell quantification (LOH) 15 days after Cas9: *SORCS1* editing (results normalized on fluorescent cell rate in non-synchronized cells). *n* = 5 independent experiments for *TP53*^WT^ cells (*n* = 3 for RO-3306 exposure) and *n* = 3 independent experiments for *TP53*^-/-^ cells. InDels were quantified by sequencing and ICE. R R0-336 (purple), P Palbociclib (orange). Mann–Whitney test used to compare two groups. **b** LOH frequency at day 15 after *SORCS1* targeting in WT cells (black), *TP53*^-/-^ cells (dark blue), *TP53*^-/-^ cells *with* p21 plasmid (light blue) and in *CDKN1A*^-/-^ cells (red, Indels in *CDKN1A* > 95%). *n* = 3 independent experiments. Mann–Whitney test used to compare two groups. **c (left)** mFAMReD system description. *Arl8b* targeting by CRISPR-Cas9 nuclease in fluorescent TdTomato^+/-^ MEFs. *Arl8b* is 5 Mb centromeric to *Rosa26* locus in mChr6. Representative cytometry analysis of non-fluorescent cells appearance (TdTom^-^) in case of LOH telomeric to the DSB. **c (right)**, Kinetic of loss of Td-Tomato fluorescence by cytometry at day 3, 7, 10 and day 14 after *arl8b* targeted by CRISPR-Cas9 in WT*, cdkn1a/p21*^*-*/-^ and *Trp53*^-/-^. NT = non-tranfected cells with background noise. Quantifications in *n* = 6 independent experiments (mean ± SD). Two-sided *T* test used to compare *cdkn1a/p21*^*-*/-^ and *Trp53*^-/-^ Cells to WT cells. Source data are provided as a Source Data file.

p53 plays a critical role in both the G1/S and G2/M cell cycle checkpoints^31^. In response to DNA DSBs, the cell cycle is arrested by activation of these cell-cycle checkpoints to facilitate DSB repair by non-homologous end-joining (NHEJ) or homologous recombination (HR). Conversely, a DSB during replication (S) and mitosis (M) could be deleterious because it can trigger replication fork collapse and induce mis-segregation of acentric fragments^32^. We hypothesized that the cell cycle phase during which the DSB occurs could influence the LOH rate. To test this hypothesis, we edited synchronized p53-proficient cells in G0/G1 and G2/M phases using palbociclib^33^, a CDK4/6 inhibitor and RO-3306^34^, a selective CDK1 inhibitor, respectively (Fig. 6a). Exposure was maintained for 24 h to 48 h after transfection to cover the Cas9 RNP activity window. RO-3306 induced a partial cell entry in the M phase and did not modify LOH frequency (Fig. 6a). In contrast, LOH frequencies were dramatically reduced (Fig. 6a) in p53-proficient and p53-deficient cells with palbociclib, which blocks cells in G0/G1 during editing (Supplementary Fig. 6a). The editing efficiency at *SORCS1* was similar to that of non-synchronized cells. Therefore, our data demonstrate that the cell cycle plays a key role in genotoxicity induced by DSB. Editing during G0/G1 limits megabase-scale LOH, even in p53-inactivated cells. Therefore, by using CDK4/6 inhibitors such as palbociclib, it is possible to prevent LOH by controlling the cell cycle.

We then tested whether p21, a well-known p53 effector and CDK inhibitor, is the missing link between p53, cell division and genotoxicity. Transient overexpression of p21 with a plasmid in *TP53*^-/-^ cells reduced LOH frequency by a third, as evaluated by the hFAMReD system at day 15 (Fig. 6b), whereas the empty control plasmid did not modify LOH frequency (1.15 ± 0.1-fold increase vs *TP53*^-/-^ cells, *n* = 4 independent experiments). This suggests that the role of p53 inactivation in genotoxicity is partially due to cell cycle deregulation. We then efficiently inactivated *CDKN1A* (p21) by CRISPR-Cas9 (97% InDels) and evaluated LOH frequency at day 15. Surprisingly, we did not observe any increase in fluorescent cells after *CDKN1A* (p21) inactivation (Fig. 6b) suggesting that cell cycle deregulation by check point inactivation is not sufficient to induce long-term LOH in p53-proficient cells. We hypothesized that LOH, which was not detected at day 15, could be induced in p21^-/-^ cells but rapidly eliminated by p53 monitoring and induced apoptosis. To observe short-term LOH, we developed an alternative system allowing LOH detection by the disappearance of Td-Tomato fluorescence. We targeted *arl8b*, which is 5 Mb centromeric to the *Td-Tomato* locus (in *Rosa26*-Chr6), in heterozygous TdTom^+/-^ primary mouse embryonic fibroblasts (MEFs). This system is named mFAMReD, for murine FAMReD (Fig. 6c). Upon LOH, fluorescent tagging can be lost. Using cytometry, we were able to detect megabase-scale LOH from day 3 (Td-Tom^neg^ cells), i.e., the delay required for Td-Tomato clearance. *Td*-*Tomato* qPCR in Td-Tom^neg^ bulk sorted by FACS confirmed *Td-Tomato* gene loss loss (Supplementary Fig. 7**)**. We created three Td-Tom^+^ MEF lines by CRISPR: WT, or inactivated for *Cdkn1a/p21*, or *Trp53*. After CRISPR DSB at *arl8b* (centromeric to *TdTomato* in *rosa26*), we monitored the dynamic of fluorescence disappearance at days 3, 7, 10 and 14 (Fig. 6c). As in hFAMReD, we found the presence of LOH in p53^-/-^ cells (1.56%, *n* = 6) at day 14, whereas it was not detectable in p21^-/-^ and WT cells. Unexpectedly, the very short-term kinetic at day 3 revealed a transient burst of Td-Tom^neg^ in both WT and p21^-/-^ cell lines. However, these LOH occurrences were lower than in p53^-/-^ cells (5.55% vs 3.85% (WT) and 2.57% (p21^-/-^)_._ The kinetic also highlighted differences in the rate of LOH decline. LOH persisted in p53^-/-^ cells whereas it rapidly disappeared in WT cells (day 7). The rate of LOH decline was slower in p21^-/-^ cells than in WT cells, and LOH was still detectable at day 7 and 10 in p21^-/-^ cells. Therefore, by using both FAMReD systems, i.e., transient p21 overexpression in p53^-/-^ cells in hFAMReD and the short-term kinetic in mFAMReD, it is possible to hypothesize that p21 participates to some extent in CRISPR-genotoxicity. Unlike p53 inactivation, p21 inactivation alone is not sufficient to maintain LOH over the long-term.

**Fig. 7:**
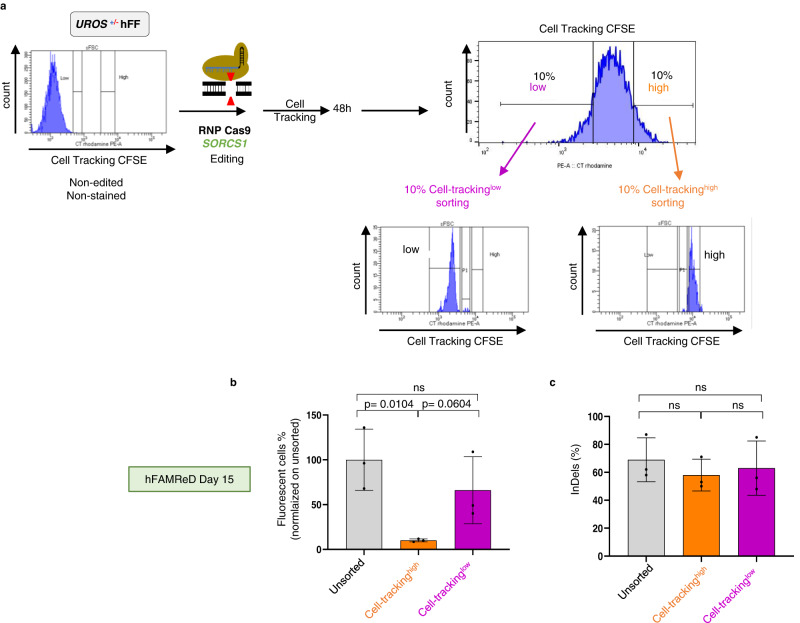
Low cell proliferation mitigates CRISPR-induced LOH frequency. **a** hFFs edited and stained with a fluorescent cell tracker. 48 h after editing, the 10% of cells with the highest MFI (“cell tracking high” cells, orange) and the 10% of cells with the lowest MFI (“cell tracking low” cells, purple) were sorted by FACS. **b**, After day 14, fluorescent cells revealing LOH are analyzed by flow cytometry in each cell fraction (mean ± SD, *n* = 3 independent experiments). **c** InDels were quantified by sequencing and ICE analysis in each cell fraction (*n* = 3 independent experiments, mean ± SD). Anova test used to compare multiple groups, Mann–Whitney or *t* test to compare two groups (two-sided). Source data are provided as a Source Data file.

To confirm the hypothesis that the cell division rate can impact LOH frequency, we stained hFAMReD hFFs with fluorescent cell tracking just before editing to monitor cell generations by dilutions. Two days post-editing, we sorted the 10% of cells with the highest mean fluorescence intensity (MFI), hereafter referred to as cell-tracking^high^ (Fig. 7a). This fraction corresponds to the cells with what may be termed a ‘low division rate’, as confirmed by a higher BrDU^neg^ fraction (Supplementary Fig. 6c). The 10% of cells with the lowest MFI (cell-tracking^low^) corresponded to the cells with the most active proliferation. Editing efficiency was similar in the three cell fractions (around 60% of InDels). At day 15 post-editing, the LOH rate of the cell-tracking^high^ fraction was dramatically reduced compared to that of unsorted (21.7-fold decrease) and cell-tracking^low^ cells (27.4-fold decrease, Fig. 7b), without impairing InDel frequency (Fig. 7c).

**Fig. 8:**
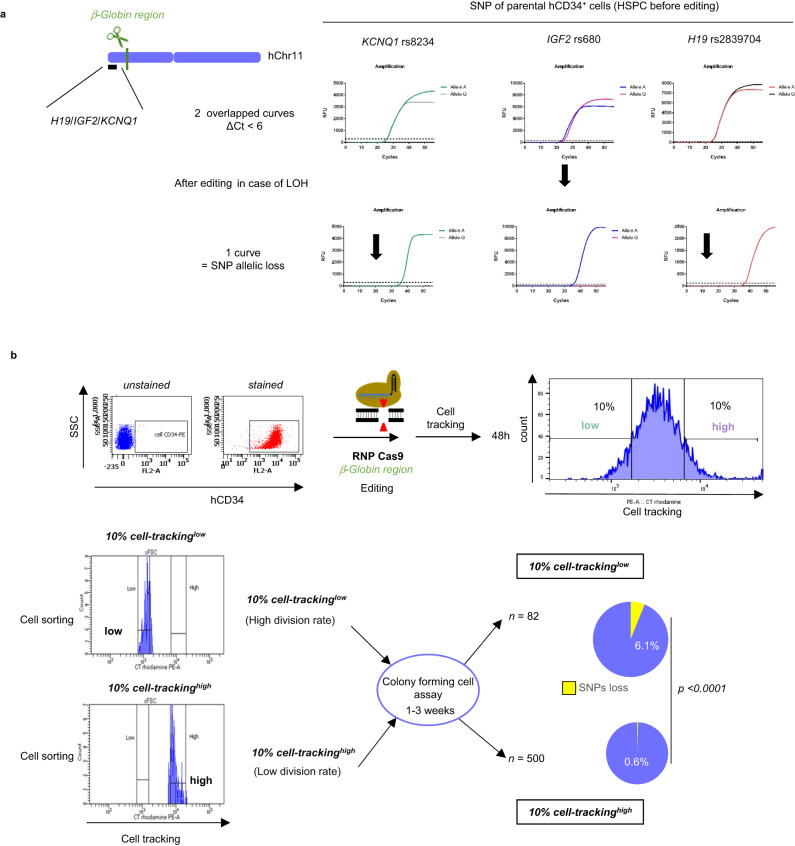
Low cell division rate prevents LOH targeting b-globin region in hCD34+ cells. **a Left** Schematic representation of globin region targeting in hCD34^+^ cells from cord blood, and *H19/IGF2/CDKN1C* imprinting center in chr11p. **Right** Identification of three SNPs by allele-specific qPCR in *H19/IGF2/KCNQ1* imprinting center, telomeric to globin region in Chr11p. Representative curves of heterozygous profile (SNP, two curves before editing in parental hCD34^+^ cells) and loss of SNP (one curve, after editing in the event of LOH (lower panel). **b** Edited hCD34^+^ cells stained with a fluorescent cell-tracker (red dye). 48 h after, the 10% highest MFI cells (10% cell tracking high, low division rate) and the 10% lowest MFI cells (10% cell tracking low, high division rate) were sorted by FACS and seeded in methylcellulose. Colonies were picked up after 1–3 weeks for SNP analysis by allele-specific qPCR. Percentage of clones with LOH (at least two SNPs loss) in low-divided clones (more clonogenic cells, *n* = 500) and in high-divided clones (less clonogenic cells, *n* = 82 clones). Chi-square test used to compare LOH frequencies.

To assess whether cell division control can prevent megabase-scale DSB-induced LOH, we targeted the clinically relevant globin cluster on Chr11p15.4 (Fig. 8a) in HSPCs. We previously reported around 1% of LOH after editing this locus^15^. Using the same protocol as described above, we analyzed the LOH rate by SNP allelic losses in HSPC subfractions sorted according to their proliferation activity. To do so, we first identified informative SNPs in the *H19/IGF2/KCNQ1* imprinting center 2.5 Mb telomeric to the cut-site (Fig. 8a, heterozygous SNP in parental cells). We considered that allelic loss in at least two telomeric SNPs was necessary to establish the existence of a telomeric megabase-scale LOH. We then sorted cell-tracking^high^ and cell-tracking^low^ edited HSPCs and performed a colony-forming cell (CFC) assay to monitor SNP genotypes at the clonal level. Despite a high InDel frequency (93%) in cell-tracking^high^ cells, single cell SNP analysis revealed a lower rate of LOH (0.6%, *n* = 500 clones) than in cell-tracking^low^ HSPCs (6.1%, *n* = 82 clones) (Fig. 8b). Taken together, hFAMReD showed that megabase-LOH genotoxicity is cell-division-dependent. Importantly, we confirmed this concept in clinically relevant cells for gene therapy protocols using CRISPR-Cas9 nuclease.

## Discussion

Successful detection, quantification, and characterization of unwanted OFF- and ON-target events after editing by nucleases are crucial for safe gene therapy. Megabase-scale ON-target genotoxicity was first described in cell lines^11,16^, and more recently in primary cells^9,13,16,17,21^, targeting different loci, with highly variable frequencies. It is unclear whether the choice of repair pathway and failures in repairing the breaks are dependent on the chromosomal DSB location and cell properties, and whether these genomic rearrangements are stable overtime. In this study, we developed two complementary FAMReD systems to study unintended terminal megabase-scale by-products induced by CRISPR-Cas9, targeting hChr10 or mChr6.

We demonstrate the ability of mFAMReD to quantify LOH early from day 3 to day 14 by the disappearance of red fluorescence. However, 0.5% of non-fluorescent MEF cells (background noise) limit the sensitivity of the method, and culture of these primary cells is limited to around six passages. Therefore, the long-term persistence of LOH cannot be studied at present by using mFAMReD. In contrast, hFAMReD is able to detect the appearance of persistent events by red fluorescence and to quantify them (only after day 15) but with higher sensitivity (0.02%) in a cell bulk. With a limit of detection of around 5–10%, FISH, aCGH, and SNP array (without subcloning/single cell analysis) all lack sensitivity to detect these rare rearrangements present in p53-proficient cells.

Although very efficient to enrich cells for LOH rearrangements, FAMReDs are limited to the analysis of hChr10q and mChr6 genotoxicity, so DSBs on other chromosomes cannot be explored. Alternatively, targeted installation of two fluorescent tags on homologous chromosome telomeres, which are lost with megabase-scale LOH, could be another quality control system to assess genotoxicity in clinical trials. Nevertheless, inversions, balanced translocations, and rearrangements centromeric to the cut-site would not be detectable. Recently, single-cell RNAseq was proposed to sensitively detect copy-loss LOH^21^. However, preponderant copy-neutral LOH was not detected and rearranged cells could not be sorted for characterization. This highlights the need for combinatorial methods to explore genotoxicity at the nucleotide (e.g., NGS, long-range PCR) and chromosome levels (e.g., CAST-seq, FAMReDs, single-cell SNP analysis) to precisely determine the genotype of edited cells.

Even though both FAMReD systems are not applicable for testing clinically relevant CRIPSR-Cas9 targets, they highlight general mechanisms to understand and prevent genotoxicity. In this study, mFAMReD made it possible to visualize a transient burst of LOH (3.85 %) induced by CRISPR-Cas9 at day 3 in WT cells. These unwanted events decreased and became undetectable at day 7. Owing to the sensitivity of this system (under 0.5%, background noise), rare events might still be present. On the other hand, the more sensitive hFAMReD system showed that some rare events persisted at day 16 and can be detected. In any case, the kinetic demonstrated that rearranged cells display a proliferative disadvantage. These data are in accordance with the Nahmad report showing a high frequency of chromosomal loss in T lymphocytes at day 4 post-CRISPR-Cas9 transfection and a 10-fold frequency decrease at day 11^21^. We observed that the frequencies of fluorescent cells were similar when targeting many loci located from 8.5 to 57.5 Mb from the telomere, suggesting that the LOH rate does not depend on the distance from the telomere. Importantly, CN-LOH predominated, regardless of the distance from the telomere, and was observed only with the most telomeric cut. We speculate that the real frequency of LOH can be obtained by doubling the observed fluorescent cell percentages (hFAMReD and mFAMReD only detect LOH by *UROS* WT and rosa26-tdTomato loss, respectively).

The mFAMReD system revealed a stronger induction of LOH by inactivation of *TP53* than in WT cells. Both systems demonstrate the persistence of LOH over time, further confirming that p53 deficiency allows the survival of genomic rearranged cells^28^. Taken together, the LOH dynamic that we observed shows that p53 is implicated both in LOH induction and maintenance. Transient p53 inhibition using two inhibitors and targeting two loci moderately increased the LOH rate. It therefore seems that enhancing gene editing efficiency by modulating p53 activity should be carried out with careful monitoring of genome integrity post-editing: p53 is a key regulator of cell cycle checkpoint and division^35–37^.

On the other hand, p21 is a key p53 effector and a major regulator of the cell cycle. Its inactivation slightly increases the short-term persistence of LOH until day 10 in the mFAMReD system compared to WT, while p21 overexpression in p53-deficient cells partially reduces LOH. p21 inactivation alone is not sufficient to maintain long-term LOH because p53 seems to remove the cells with LOH in both p21-deficient cells and WT cells.

Good timing in the cell cycle is known to be important when attempting to understand and improve editing. While NHEJ is known to be active throughout the cell cycle, HR is the most active during the S phase. Here, we highlight the impact of the cell division rate on genotoxicity during editing in hFFs and HSPCs. G0/G1 synchronized cells and low-divided cells were protected from LOH. Cells may be protected by enhanced canonical NHEJ^38,39^. Indeed, the c-NHEJ repair pathway could limit LOH by joining both chromosomic parts after DSB. This would be in accordance with two studies^40,41^ showing that when cNHEJ is deficient, DSBs are repaired by genotoxic pathways and induce kilobase-scale rearrangements. Here, we demonstrate that a low proliferation rate strongly reduces the occurrence of LOH without compromising NHEJ editing efficiency (in two human cell models, targeting two different chromosomes and using two methods to quantify LOH). The relative risk of megabase-scale LOH induced by CRISPR is reduced 10-fold in hCD34^+^ cells with a low division rate. Importantly, cell cycle arrest by palbociclib was able to suppress genotoxicity even in p53-deficient cells. This concept should be considered when designing clinical cell culture protocols before editing. Slowing down cell proliferation before editing could decrease this ON-target genotoxicity.

Our data show that FAMReDs provide versatile platforms to identify solutions to reduce CRISPR-related LOH. They could be use to screen chemical libraries to find drugs that prevent ON-target genotoxicity and to evaluate the genotoxicity of other CRISPR tools (DSB-free genome editors like prime editing^42^, base-editors^43^, or double-spacer nicking^44^).

By leveraging the phenotype switch, FAMReDs offer another advantage in the possibility of isolating cells with stable LOH for in-depth analysis. By using hFAMReD, we isolated rearranged cells and observed different types of LOH, (CL-LOH or CN-LOH), starting from the cut-site to the telomere. Interestingly, the rearrangement type profile (CN-LOH/CL-LOH ratio) was distinct when targeting different loci. In all cases, CN-LOH was predominant. We only observed CL-LOH in the event of DSB at the closest target to the telomere (8.5 Mb). Notably, the rearrangement type profile was not modified by *TP53* inactivation for a defined locus. Further studies will be required to evaluate whether CN-LOH, which is not detectable by FISH or aCGH, has a functional impact, depending on the genes in the chromosomal region of interest. This CN-LOH is probably due to a loss of chromosome extremity and to the secondary duplication of the remaining allele by break-induced replication (BIR)^45^ to avoid CNV. Such large LOH could contribute to tumorigenesis by activating potential oncogenes or by unmasking mutated tumor suppressor genes.

In this study, LOH was sometimes associated with centromeric extra-large duplication, again illustrating the diversity of ON-target rearrangements induced by CRISPR-Cas9 nuclease. Complex large kilobase-scale rearrangements have already been reported^6,10^. The mechanisms of this unexpected rearrangement linking duplication with terminal LOH remain elusive. It could be due to a U-type exchange, a breakage-fusion-bridge process^46^, non-allelic HR, a fold-back mechanism^47^, or possibly chromothripsis^17,48^.

In conclusion, we have developed two cytometry-based megabase-scale LOH detection systems (FAMReDs). They revealed and quantified the genotoxicity of CRISPR-Cas9 in primary cells with high sensitivity. Importantly, we identified mechanisms linking p53, cell division and genotoxicity. Cell cycle blockade by palbociclib prevented ON-target megabase-scale genotoxicity without compromising the efficacy of NHEJ. These data offer opportunities to make nuclease-based gene therapy protocols safer.

## Methods

### Ethical statement

Our research complies with all relevant ethical regulations. Human CD34^+^ HSPCs were isolated from the cord blood of healthy donors from Bagatelle Hospital, according to the hospital’s ethical institutional review board (Maison de Santé Protestante de Bordeaux, Talence, France) and with the mother’s informed consent. Mice were produced and housed at the University of Bordeaux animal facility, according to the rules and regulations of the Institutional Animal Care and Use Committee (agreement no. A33-063-941).

### Cell culture

Human foreskin fibroblasts immortalized with *hTERT* (hFF *hTERT*) used in hFAMReD system were from ATCC® (CRL 4001, BJ-5ta). They were partially inactivated for *UROS* and totally inactivated for *TP53* using an RNP made of Cas9 protein complexed with a gRNA targeting *UROS* exon 4 and *TP53* exon 4, respectively.

Primary MEFs heterozygous for TdTomato in *Rosa26* (mChr6), used in the mFAMReD system, were obtained in our laboratory. Briefly, we crossed homozygous Td-Tomato^+/+^ male mice (Jackson laboratory #007576), with WT female C57Bl6/j mice to obtain embryos heterozygous for TdTomato^+/-^. At day 13.5, embryos were retrieved and dissected for MEF culture. Primary MEFs were cultured for six passages. Cell parts were inactivated for *p53* and *cdkn1a* using CRISPR-Cas9 RNP.

hFFs and MEFs were maintained in Dulbecco’s modified Eagle’s medium (DMEM with glucose (4.5 g.L^−1^), L-Glutamine (1 g.L^−1^) and pyruvate supplemented with 20% fetal bovine serum, MEM non-essential amino acids 100X (Gibco® by ThermoFisher scientific, Carlsbad, CA, USA), 100 U/mL penicillin, and 100 μg/mL streptomycin (all from Eurobio, Courtaboeuf, France). 0.1 mM Beta-mercaptoethanol (Gibco® by ThermoFisher scientific, Waltham, Massachusetts, USA) was added to the MEF medium.

To inhibit p53 activity transiently, we added pifithrin-alpha (30 µM, 15 h before transfection and for 5 days after transfection) in the medium.

Human CD34^+^ HSPCs were isolated from the cord blood. Briefly, mononuclear cells were isolated by Ficoll gradient. CD34^+^ cells were purified according to the manufacturer’s instructions (Human CD34-Positive Selection kit II ref 17865 from Stem Cell Technologies) and purity was analyzed by flow cytometry using PE-conjugated anti-CD34 antibody (clone 561 Biolegend (San Diego CA, USA) 25 μg/mL, lot # B2044487, 5 μL/test). Cryopreserved CD34^+^ cells were thawed and cultured in expansion medium consisting in Stem Span SFEM (Stem Cell Technologies) supplemented with hFlt3-L (50 ng/mL), SCF (50 ng/mL), human TPO (50 ng/mL), human IL3 (20 ng/mL) and human IL6 (10 ng/mL) (all from Peprotech), StemRegenin 1 (SR1) (1 μM) (Stem Cell Technologies), VPA valproic acid (500 µM) (Sigma-Aldrich), 100 U/mL penicillin, and 100 μg/mL streptomycin (Eurobio). Two days after thawing, CD34^+^ cells were transfected with Cas9 RNP (see below). After cell tracking staining and sorting, CD34^+^ cells were plated in 35 mm tissue culture dishes at 50 and 1000 cells/mL with 1 mL of methylcellulose medium (Stemcell Technologies, MethoCult H4034 Optimum). From 1 week, individual colonies were subsequently picked from plates and washed in PBS to remove all the methylcellulose. Cells were digested with proteinase K in lysis buffer (10 mmol/L Tris–Cl, pH 8.0, 50 mmol/L KCl, 2.5 mmol/L MgCl_2_, 0.5% Tween 20, 100 mg/mL proteinase K) at 56 °C for 1 h, followed by a 10 min exposure at 95 °C.

All cells were cultured in a standard humidified 37 °C, 5% CO_2_ incubator.

### Transfection and gene editing tools

Cells were transfected by electroporation using the AMAXA™ 4D-Nucleofector™ device (Lonza®, Bale, Switzerland) with P3 Primary Cell Line and CZ-167 and D0-100 programs for fibroblasts (murine and human) and hCD34^+^ cells, respectively. In brief, 200,000 cells were nucleofected with 16.9 μg Cas9 RNP and 5 μM of Alt-R® Cas9 Electroporation Enhancer. To form RNP, Alt-R® S.p.Cas9 Nuclease V3 protein (or either HiFi Cas9, Cas9^D10A^ or dCas9 when specified) was complexed to crRNA:tracrRNA according to the manufacturer’s instructions. Then complexes were incubated for 20 min at room temperature before electroporation. Cas9 proteins and crRNA were purchased from Integrated DNA Technologies, Coralville, USA. All gRNA sequences are in Supplementary Table 2.

pcDNA3 MDM2 WT was a gift from Mien-Chie Hung (Addgene plasmid # 16233; RRID:Addgene_16233)^49^. pcDNA3 backbone was used as a plasmid control. They were transfected by nucleofection 24 h before editing by CRISPR-Cas9.

To overexpress human p21 in hFFs, we transfected the “flag p21 WT plasmid” (p21 plas.) by nucleofection 24 h before transfection of CRISPR-Cas9. Flag p21 WT was a gift from Mien-Chie Hung (Addgene plasmid # 16240; http://n2t.net/addgene:16240; RRID:Addgene_16240)^50^.

### InDels quantification

Genomic DNA of edited cells, and their associated controls, was extracted using Nucleospin® Tissue (Macherey-Nagel®) according to the manufacturer’s protocol. The genomic region flanking the expected cut-site was amplified by PCR (HotStarTaq Plus DNA polymerase, Qiagen®, Venlo, Netherlands) with adequate primers (Supplementary Table 3). PCR products were purified with Nucleospin® Gel and PCR Clean-up (Macherey-Nagel). Sanger sequencing was done on purified PCR products and sequenced by LIGHTRUN (GATC Biotech, Konstanz, Germany). Sanger sequencing data were analyzed using ICE v2 CRISPR Analysis tool (ICE) software (Synthego, Redwood City, USA). Purified PCR products from non-edited cells were used as control chromatogram.

### Cell cycle analysis and synchronization

Cell cycle analyses were performed to check synchronization efficiency. Briefly, cells either synchronized or not were harvested and washed twice with PBS, then fixed with 70% ethanol in PBS overnight at 4 °C. Cells were washed twice with PBS and incubated with a mix containing RNAse (1 mg/ml) and PBS-propidium iodide (0.5 μg/ml, for 15 min), both from Sigma-Aldrich, Saint Louis, USA. The samples were examined on a BD Biociences Accuri C6 Plus apparatus and the data were analyzed with BD CSamplerTM software (BD Biosciences, Le Pont de Claix, France).

To evaluate the impact of synchronization on the occurrence of LOH, cells were synchronized in G0/G1 phase by incubation with palbociclib, also named PF-00080665 or PD 0332991 (1 µM, Sigma Aldrich) 24 h before and 24–48 h after RNP transfection (Supplementary Fig. 6). To synchronize cells in the G2/M phase, cells were incubated with RO-3306 (10 µM, Sigma Aldrich) for 24 h before RNP transfection and 24 after RNP transfection.

### Proliferation assays

Cell tracking. Edited cells (hFFs or hCD34^+^ cells) were stained immediately after editing. To do so, cells were plated and stained for 3 h with 5 µL of Cell Tracking Red Dye Kit (Abcam, reference ab269446, Cambridge, UK) per 100 µl of medium. After 3 h of incubation in the staining medium, cells were PBS-washed three times by centrifugation at 500 g for 10 min. After 48 h, cells were FACS-sorted to isolate 10% cell tracking^high^ and 10% cell tracking^low^ cells and expanded (FACS Aria, BD). Comparison of initial and 48 h cell tracking curves was performed using FlowJo Software (BD biosciences).

Cell count. To define the cell proliferation difference between the cell tracking^high^ and cell tracking^low^ cells, both fractions were compared. Immediately after sorting and 4 days post-sorting, cells were counted manually using the KOVA Glasstic Slide 10 with Grid (KOVA™ 87146, Fisher Scientific, Waltham, MA, USA). Simultaneously, cell numbers were also defined by using a Luna IITM Automated Cell Counter (Logos Biosystems, South Korea).

BrdU staining. Cell tracking^high^ and cell tracking^low^ cells were incubated with 10 µM bromodeoxyuridine or BrdU (FITC BrdU Flow Kits from BD Pharmingen™, BD BioSciences, Le Pont de Claix, France) for 20 and 48 h. Cells were then resuspended in 100 µL of BD Cytofix/Cytoperm Buffer and incubated for 15 to 30 min on ice. After fixing, cells were washed by adding 1 mL of BD Perm/Wash Buffer by centrifugation at 200 to 300 g for 5 min. The supernatant was discarded. To permeabilize the cells, 100 µL of BD Cytoperm Permeabilization Buffer Plus were added and incubated 10 min on ice. Cells were treated with DNase (30 μg /10^6^ cells) and incubated for 1 h at 37 °C. Afterwards, BrdU was stained with fluorescent antibodies for 20 min in the dark at room temperature. After the final wash, cells were resuspended in 1 mL of Cell Staining Buffer to be analyzed by flow cytometry (BDFACS CantoTM, BD).

### hFAMReD fluorescent cell quantification and sorting

At day 15 post-editing, 0.3 mM of 5-ALA were added to hFF media. After 16 h of exposure (overnight), cells were washed twice with 1X PBS and put back in fresh media. Upon LOH, loss of *UROS* can be detected by the appearance of fluorescence due to porphyrin accumulation. Following 8 h of clearance, fluorescent cells were quantified by flow cytometry (Supplementary Fig. S1b). UV-sensitive porphyrins were excited at 488 nm and the emitted wavelength was approximately 667 nm, detected by the PE-Cy5A PMT channel (FACS Accuri, BD, Franklin Lakes, NJ, USA). FL-1 is a control green-fluorescent channel used to exclude auto-fluorescent cells. Fluorescent-positive or -negative fractions were sorted by BD FACS Aria®.

### mFAMReD non-fluorescent cell quantification

We edited the *arl8b* locus (5 Mb centromeric to *TdTomato* insertion in *Rosa26*, in mChr6) in fluorescent heterozygous TdTomato^+/-^ MEFs. At day 3, 7 10 and 14 post-editing, upon LOH, loss of Td-Tomato can be detected by the disappearance of fluorescence.

### SNP analysis by Sanger sequencing of *DOCK1/MGMT/UROS* and by allele-specific quantitative PCR in 11p15 region

We used dbSNP (NCBI) to screen frequent SNPs in parental cells. In fluorescent hFFs, we tested SNPs in *UROS*, *DOCK1* and *MGMT* by Sanger sequencing to confirm Chr10q LOH. The genomic regions flanking SNPs were amplified by PCR (HotStarTaq Plus DNA polymerase, Qiagen®, Venlo, Netherlands) with adequate primers (Supplementary Table 4). PCR products were purified with Nucleospin® Gel and PCR Clean-up (Macherey-Nagel) and sequenced.

For the methylcellulose CFC assay, we selected and tested SNPs in *H19*, *IGF2* and *KCNQ1* to screen Chr11p LOH. SNP genotyping was performed by real-time quantitative PCR analysis (CFX Connect device, Biorad®) of genomic DNA with a common reverse primer and two SNP allele-specific forward primers. Only curves with Ct < 37 were included for the analysis. To be considered as an SNP loss (homozygous), the profile can be only one or two curves with a delta Ct  > 6.

### Array CGH and SNP array

aCGH was performed on 8 × 60 k oligonucleotide microarrays (Agilent Technologies, CA). DNA was labeled (cyanine 3 or cyanine 5) using the Genomic DNA ULS Labeling Kit from Agilent Technologies and hybridized onto the microarrays according to the manufacturer’s instructions (Agilent). Scanning of the microarrays was performed using a G5761A scanner (Agilent). Data analysis was carried out with Agilent Technologies software, namely Feature Extraction for Cytogenomics V5.0 to calculate the fluorescence ratio and Agilent CytoGenomics 5.2 to visualize chromosomal imbalances. Deletions and duplications in the heterozygous state were characterized by values of the log2 ratio of fluorescence intensities (cyanine5/cyanine3) below −0.5 and above +0.3, respectively, with the statistical algorithm ADM2 used at a threshold of 5.

Combined SNP/CGH arrays was performed on Genetisure Cyto 180 K CGH/SNP arrays (Agilent Technologies, Santa Clara, USA). DNA was labeled (cyanine 3 or cyanine 5) using the Genomic DNA ULS Labeling Kit from Agilent Technologies and hybridized onto the microarrays according to the manufacturer’s instructions (Agilent). For SNP/CGH arrays, tested DNAs were hybridized against male control DNA obtained from Agilent. Microarrays were scanned with a G5761A scanner (Agilent). Data analysis was carried out with Agilent Technologies software, namely Feature Extraction for Cytogenomics Algorithm V5.0.1.16 to calculate the fluorescence ratio and Agilent CytoGenomics 4.0 and 5.0 to visualize chromosomal imbalances and LOH. Deletions and duplications in the heterozygous state were characterized by values of the log2 ratio of fluorescence intensities (cyanine 5/cyanine 3) below −0.5 and above +0.3, respectively, with the statistical algorithm ADM2 used at a threshold of 5. LOH was evaluated with the statistical algorithm ADM2 used at a threshold of 6 (Default Analysis Method v2).

For Supplementary Fig. 4, combined SNP/CGH arrays were performed with Illumina Infinium CytoSNP-850K BeadChip and Nextseq 550 array scanning system according to the manufacturer’s protocol (Illumina). Specific beadchip DMAP files were loaded with Decode File Client software (Illumina) and data were analysed with Blue Fuse Multi v4.5 software (Illumina).

### Statistics and reproducibility

All experiments were conducted at least time independently. No statistical method was used to predetermine sample size. No data were excluded from the analyses. The experiments were not randomized. Cytogenetic analysis (CGH array and SNP array) was performed blinded. Statistical significance was inferred when necessary. Exact distinct and independent experiments size is indicated in each legend (*n*). Graph Pad Prism 6 software was used for statistical analysis. Results are presented as mean ± SD. The parametric *T* test (two-sided) was used when distribution was Gaussian/normal (Shapiro–Wilk test). The non-parametric Mann–Whitney test (two-sided) was used to compare two groups. One-way ANOVA (two-sided), complemented with the unprotected Fisher’s Least Significant Difference test, was used to compare more than two groups. Percentages of LOH in HSPC were compared by the Chi-square test.

### Reporting summary

Further information on research design is available in the Nature Portfolio Reporting Summary linked to this article.

## Supplementary information

Supplementary Information

Reporting Summary

## Data Availability

Source data are provided with this paper. The source data generated in this study have been deposited in the ZENODO database 10.5281/zenodo.8063612. The data discussed in this publication have been deposited in NCBI’s Gene Expression Omnibus (Edgar et al., 2002) and are accessible through GEO Series accession number: GSE235019, GSE235482, GSE235485, GSE235487, GSE235488, GSE235491. Source data are provided with this paper.

## Source data

Source Data
