# Supplementary material for: Cell cycle arrest and p53 prevent ON-target megabase-scale rearrangements induced by CRISPR-Cas9
Source: Nat Commun. 2023 Jul 10;14:4072. doi: 10.1038/s41467-023-39632-w (PMC10333231; doi:10.1038/s41467-023-39632-w)
Supplement: Supplementary file 1 — Supplementary Information [file 41467_2023_39632_MOESM1_ESM.pdf]

Supplementary Fig. 1

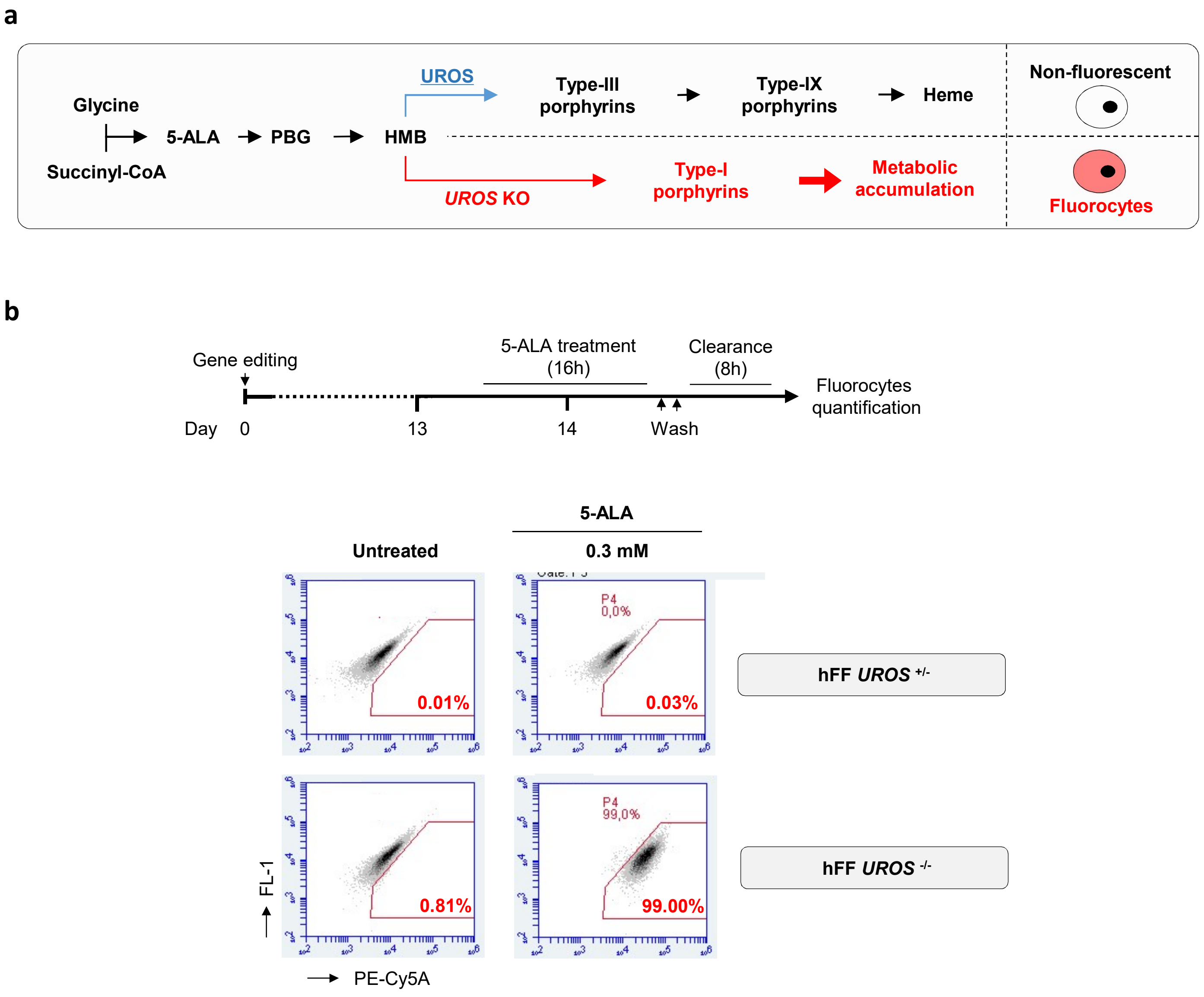

Supplementary Fig. 1. Heme biosynthesis and porphyrins accumulation. (a) Scheme of the heme biosynthesis. When UROS enzyme is functional, it leads to heme biosynthesis trough type-III and type-IX porphyrins formation. When UROS enzyme is totally impaired in erythroid lineage, HMB spontaneously forms type-I porphyrins that cannot continue biosynthesis pathway (metabolic dead-end). If sufficient level of type-I porphyrins accumulates, it turns the cell into fluorocytes. 5-ALA: 5-amino-levulinic acid; PBG: porphobilinogen; HMB: hydroxymethylbilane. UROS: uroporphyrinogen-III synthase (EC 4.2.1.75). **(b)** Detection of UROS impairment by cytometry in fibroblasts required a transient and low exposure to 5-ALA (0.3mM, overnight) followed by an 8h-clearance. Illustrative cytometry: *UROS*$^{+/-}$ fibroblasts, used in FAMReD, have residual UROS enzymatic activity and are not fluorescent, even with 5-ALA exposure. In contrast, *UROS*$^{-/-}$ fibroblasts, have no residual UROS activity and are fluorescent.

Supplementary Fig. 2

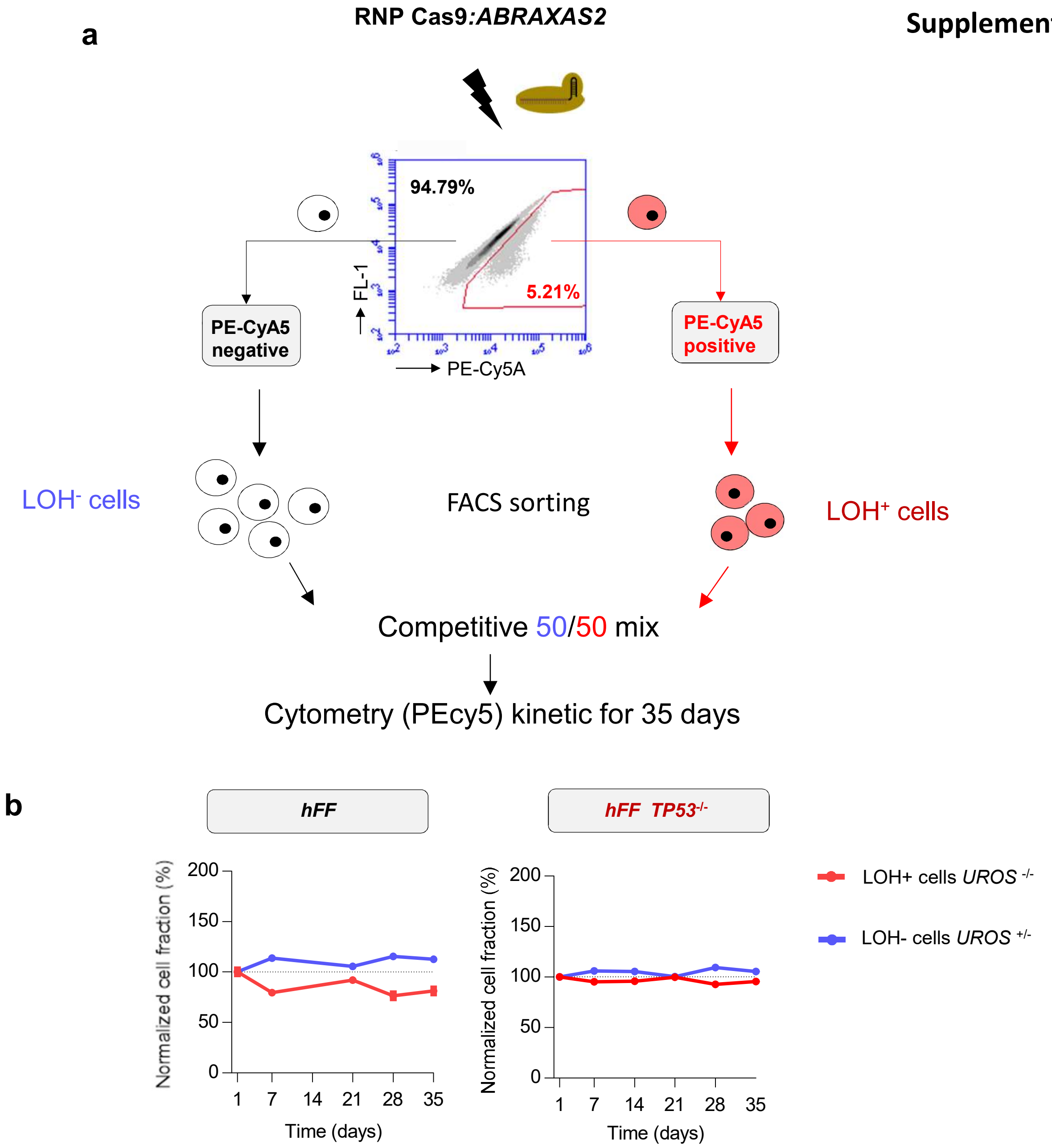

Supplementary Fig. 2. LOH appearance, with *UROS* homozygous mutations, does not impair cell proliferation. **a,** To affirm that proliferation of cells with LOH (*UROS*$^{-/-}$) is not disturbed, we performed a sorting of LOH^{+} cells (*UROS*$^{-/-}$ in red) and LOH^{-} heterozygous *UROS*$^{+/-}$ cells (in blue) and compare their proliferation by a competitive assay (mix 50:50) for 35 days. **b**, We did not observe selective advantage of one population, neither in WT fibroblasts (n = 3 independent experiments) nor in *TP53*$^{-/-}$ fibroblasts (n = 2 independent experiments) . LOH cells are stable in long-term culture.

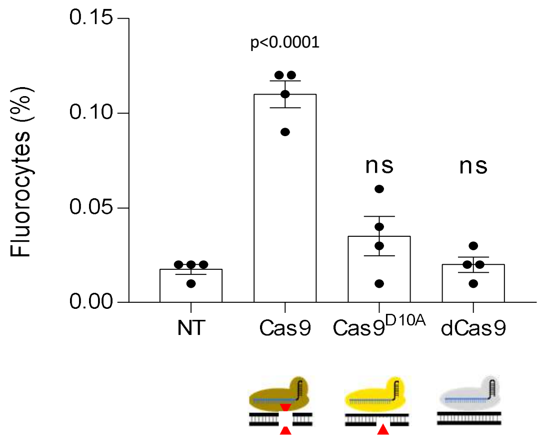

Supplementary Fig. 3: Example of FAMReD application. Fluorocytes quantification in not transfected (NT) or transfected hFF with either Cas9 nuclease, Cas9^{D10A} nickase or dead Cas9 (dCas9) complexed with a gRNA targeting *ABRAXAS2*, (mean±SD, n=4 independent experiments). One way Anova test used to compare two groups.

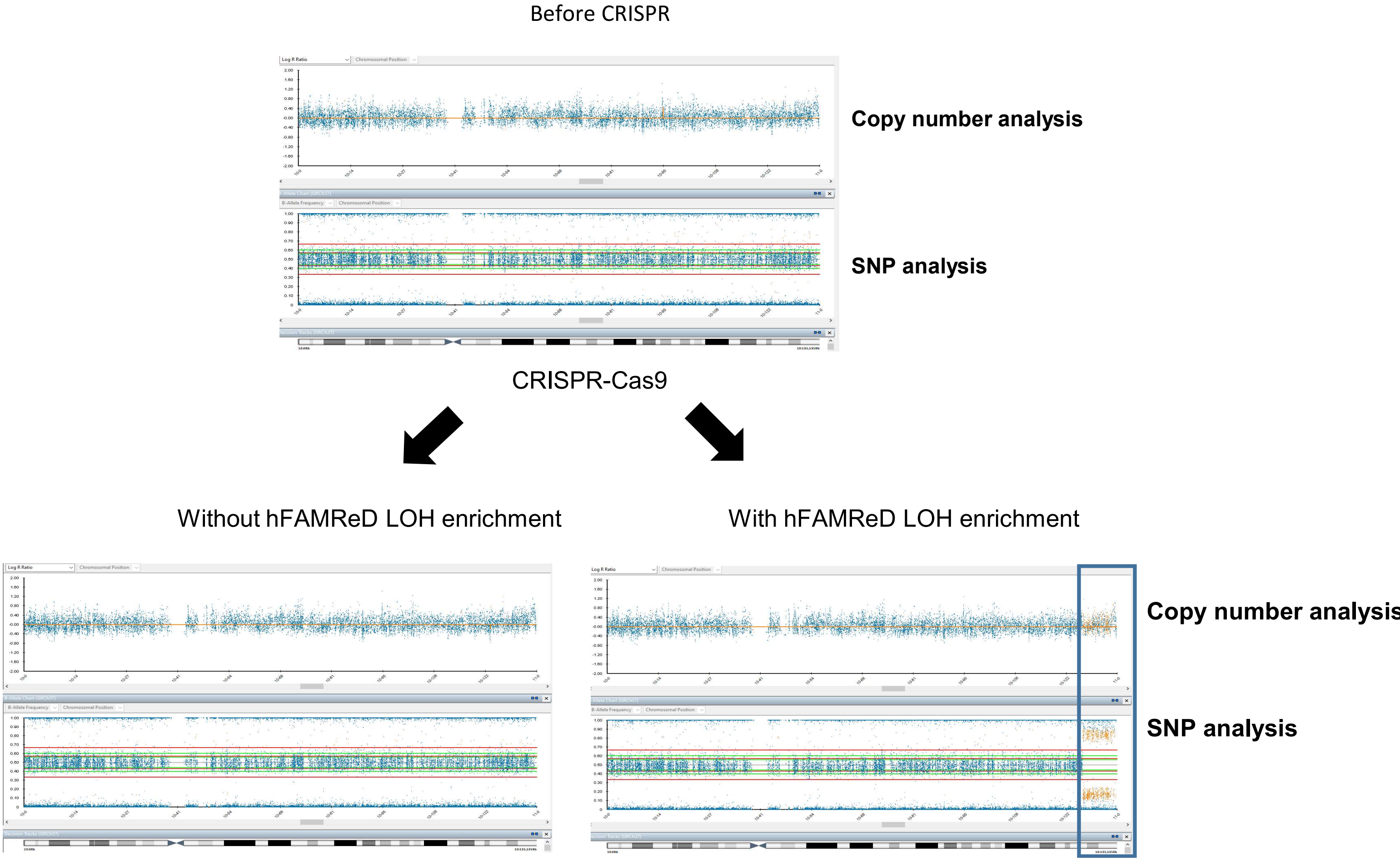

Supplementary Fig. 4. hFAMReD is required to reveal LOH induced by CRISPR targeting *ABRAXAS2* in WT hFF. LOH are not detectable by cGH/SNP array without FAMReD system (under limit of detection) and detected after hFAMReD cell sorting enrichment.

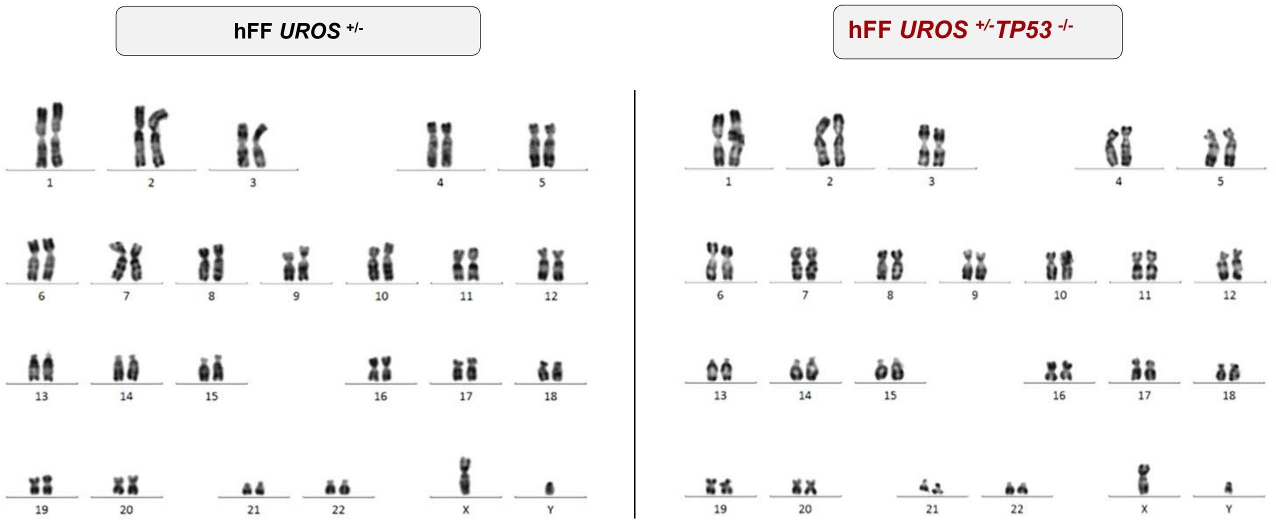

Supplementary Fig. 5. Karyotype of generated fibroblast cell lines: before (left panel) and after *TP53* invalidation by CRISPR-Cas9 (right panel). The two cell lines maintained a normal karyotype in all analyzed nuclei.

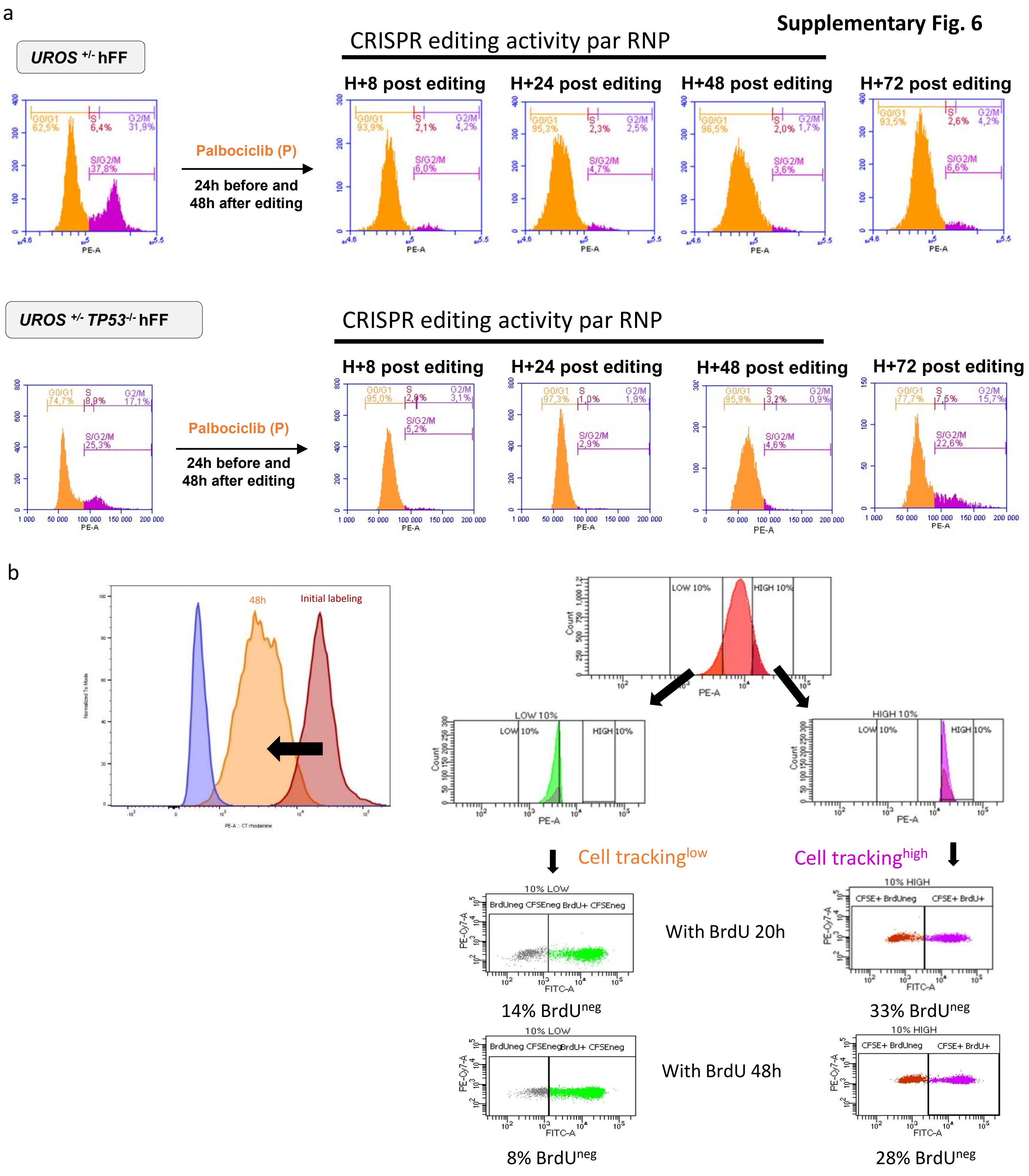

Supplementary Fig 6. Cell division modulation. **a**, Kinetic of G0/G1 blockade by palbociclib in WT and p53 KO hFF cells. 72h after editing, cells re-engage in the cell cycle. **b**, cell tracking analyze after 48h. We observed a shift of fluorescence between the initial labeling (in red) and 48 hours (in orange). Mean of fluorescence decreased from 26000 to 4000. Unlabeled cells in blue. BrdU incorporation in cell tracking^{high} and cell tracking^{low} fractions during 20h and 48h. Cell tracking^{high} is enriched in BrdU^{neg} cells confirming a lower division rate (one experiment).

		qPCR TdTomato		qPCR b actin		Tomato/b actin	copy number
		Ct	Delta Ct	Ct	Delta Ct		
cell sorting #1	Tomato + MEF	20.86	6.29	16.76	1.95	0.058314562	1
	Tomato - MEF	27.15		18.71		0.002879432	0.049
cell sorting #2	Tomato + MEF	24.32	4.59	18.13	0.19	0.013696964	1
	Tomato - MEF	28.91		18.32		0.000648772	0.047

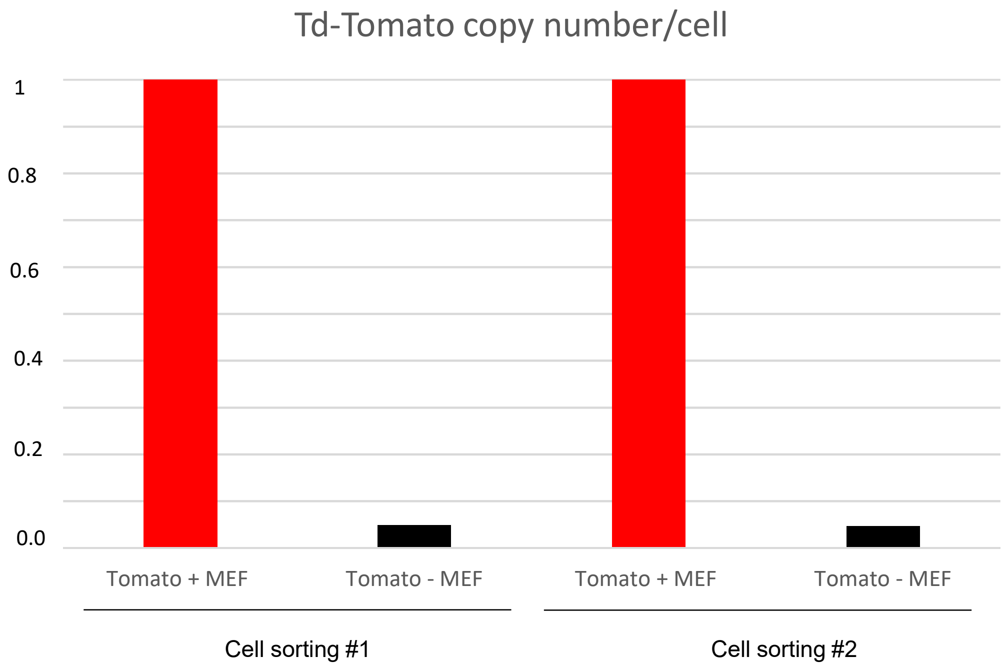

Supplementary figure 7: qPCR of *Td-Tomato* transgene in MEF cells after cell sorting of *Td-tomato*^{pos} and *Td-tomato*^{neg} by cytometry, to demonstrate genomic *TdTomato* loss and LOH telomeric to *Arl8b* after cut by CRISPR-Cas9 nuclease.

Supplementary Table 1: predicted OFF-target sites by IDT website and CRISPOR software

Séquence	PAM	Locus OFF-T IDT	
GAGATCCTCCCACTCGATGG	AGG	ABRAXAS2: target	Chr10q:-124829881
TTGATCTTCCCACTCAATGG	AGG	Off-target 1	Ch13:-87362303
GGCATCCTCCCATTCAATGG	CAG	Off-target 2	Chr6:-166445710
GATGTCATCCCACTCAATGG	TGG	Off-target 3	Ch15:+76145921
AAGATCAGCCCACTAGATGG	AAG	Off-target 4	Chr6:+6923378
GAGGTCTTCTCACTCGATGC	TGG	Off-target 5	Chr16:+11640559
GAGAGCCCCCCACTCAGTGG	GGG	Off-target 6	Chr20:+62663672
CAGATCCCACCACTCAATGG	GAG	Off-target 7	Chr3:+99808837
CAGACCCTCCAACCCGATGG	AAG	Off-target 8	Chr10p:+21820668
TATATCCTGCCACTCGATGG	GAG	Off-target 9	Chr3:-11162298
GAGAACCACCCACACGATGG	CAG	Off-target 10	Chr3:-16129034
**Séquence**	**PAM**	**Locus OFF-T CRISPOR**	
GAGATCCTCCCACTCGATGG	AGG	ABRAXAS2: target	Chr10q:-124829881
TTGATCTTCCCACTCAATGG	AGG	Off-target 1	Chr13:-88014556
GAGGTCATCACACTCGAAGG	AGG	Off-target 2	Chr16:+7294761
GATGTCATCCCACTCAATGG	TGG	Off-target 3	Chr15:+76438263
GAGGTCTTCTCACTCGATGC	TGG	Off-target 4	Chr16:+11734416
GAGATTCTCCAACTAAATGG	CGG	Off-target 5	Chr3:+142747326
AAAATCCTTCCACTAGATGG	CGG	Off-target 6	Chr18:+39694473
GGGCTGCTCACACTCGATGG	TGG	Off-target 7	Chr13:-113473682
GAGAGCCCCCCACTCAGTGG	GGG	Off-target 8	Chr20:+61295025
GTGACCCTCCCACTCATTGG	AGG	Off-target 9	Chr3:-194600571
GAGAACTTCCCACTCACTGG	GGG	Off-target 10	Chr14:-70161749

Supplementary Table 2: gRNA sequences for CRISPR-Cas9 editing.

Guide RNA sequences	
UROS	GGAAGCAGCAGAGTTATGTT
TP53	CCATTGTTCAATATCGTCCG
ABRAXAS2	GAGATCCTCCCACTCGATGG
SORCS1	TGATAGACGGTGTGCCGAAG
b-Globin region 1	ACCAATAGAAACTGGGCATG
b-Globin region 2	AGGGTGCTACATACTTCCTA
CPXM2	GCCTCATGACAGACGCCCGG
PLEKHA1	CTGGCGCCATTGTAGCACAG
TRUB1	ATACACTAGATTCTACGGGG
ADRA2A	TGGTCGTTGATCTCGCAGCG
VCL	CGTATGAAACACTGGCATCG
CDKN1A (p21)	GTCGAAGTTCCATCGCTCAC
Murine *cdkn1a* (p21)	GCGCAACTGCTCACTGTCCA
Murine *Trp53*	ACTCCAGGTAGGAAGGCGCG
Murine *Arl8b*	AGATGGAACTGACGCTCGTG

Supplementary Table 3: Primers for PCR and Sanger sequencing (for further indels quantification by ICE).

Primers PCR		
Indels analysis		Target cells
UROS F	TAGTTCCAGGCACATAGTAAGCAC	Human Fibroblasts
UROS R	AGGAGGTGAACAACGAATAGACAG	
TP53 F	TGGTCCTCTGACTGCTCTTTTC	Human Fibroblasts
TP53 R	GGAAGCCAAAGGGTGAAGAGG	
ABRAXAS2 F	TGATGAAAGACATCAGGGCGA	Human Fibroblasts
ABRAXAS2 R	AAGAGCGTTTGAACTGGCCT	
SORCS1 F	TTGGATCTGAGTGCTGAACTGG	Human Fibroblasts
SORCS1 R	TGAACGCCCCACAAATGCTC	
b-Globin region F	AGCACCGCCTATCTATGTGC	HSPC
b-Globin region R	GGAAACTGGATGCAGAGACCA	
CPXM2 F	GAGACCAGAGCAGTCATAGCCT	Human Fibroblasts
CPXM2 R	GACAGTGCCCTCCTCCTTCT	
PLEKHA1 F	TTAGGCTGATAGCCCTGAAGAG	Human Fibroblasts
PLEKHA1 R	AATTTGATAGGATGGGGGAGAC	
TRUB1 F	TGCATAACAGTTTTTGTTTGCC	Human Fibroblasts
TRUB1 R	TAGCCCAAAGAAACACACTGAG	
ADRA1 F	ACACAGGCCATCGAGTACAAC	Human Fibroblasts
ADRA1 R	CTGGTAGATGCGCACGTAGAC	
VCL F	CCGTGGATCCTACTTCTCTGTC	Human Fibroblasts
VCL R	GTGAGGTCAGGAATGGCTTTG	
CDKN1A/p21 F	GCGACTGTGATGCGCTAAT	Human Fibroblasts
CDKN1A/p21 R	CAAGACAGTGACAGGTCCACAT	
Murine *Trp53* F	CGGCTCTGAGTATACCACCATC	MEFs
Murine *Trp53* R	CAAGAGGAAACAGAGGAGGAGA	
Murine p21 F	CTTAGTCTCATGGTGTGGTGGA	MEFs
Murine p21 R	GAAGTCAAAGTTCCACCGTTCT	
Murine *arl8b* F	AAAGAGTGTCCGCTGTCGTC	MEFs
Murine *arl8b* R	GCGATGACATTGACGAAGGT	

Supplementary Table 4: Primers for SNP analysis and qPCR.

**Primers PCR and AS-PCR**			
Target loci	Primer sequences		Target cells
DOCK1<br>*rs867002*<br>*rs867003*	DOCK1 F	ACCTACCGGCGATCATGAAG	Fibroblasts
	DOCK1 R	CCAACCCCTGTTCTCACACA	
MGMT<br>*rs511361*<br>*rs1846361104*	MGMT F	TCTCCCACTGTTGGTCGTTG	Fibroblasts
	MGMT R	GGATCCCTTTTCCGGGTCTC	
KCNQ1<br>*rs8234*	KCNQ1 #2 A F	GGGTTCCTTCTGGGCATCACA	HSPC
	KCNQ1 #2 G F	GGGTTCCTTCTGGGCATCACG	
	KCNQ1 #2 common R	CAGGAACCAAGGTGAGAGCAGT	
H19<br>*rs2839704*	H19 A F	GAGACGGCCTTGAGTCTCGGTA	HSPC
	H19 G F	AGACGGCCTTGAGTCTCGGTG	
	H19 common R	CTTGAAGGCTGCTCCGTGATGT	
IGF2<br>*rs680*	IGF2 A F	GAACCAGCAAAGAGAAAAGAGGGA	HSPC
	IGF2 G F	AACCAGCAAAGAGAAAAGAGGGG	
	IGF2 common R	GAGCCAGTCTGGGTTGTTGCTA	
tdTomato	tdTomato F	CAACTGCCCGGCTACTACTAC 3’	MEF
	tdTomato R	GACGGCCATGTTGTTGTCCTC	
Beta-actin	Beta-actin F	CATTGCTGACAGGATGCAGAAGG	MEF
	Beta-actin R	TGCTGGAAGGTGGACAGTGAGG	
